# Accurate Cure Modeling for Isothermal Processing of Fast Curing Epoxy Resins

**DOI:** 10.3390/polym8110390

**Published:** 2016-11-03

**Authors:** Alexander Bernath, Luise Kärger, Frank Henning

**Affiliations:** 1 Karlsruhe Institute of Technology, Institute of Vehicle System Technology, Chair for Lightweight Technology, Rintheimer Querallee 2, 76131 Karlsruhe, Germany; luise.kaerger@kit.edu (L.K.); frank.henning@ict.fraunhofer.de (F.H.); 2 Fraunhofer Institute for Chemical Technology, Joseph-von-Fraunhofer Str. 7, 76327 Pfinztal, Germany

**Keywords:** reaction kinetics modeling, differential scanning calorimetry, fast curing resin, epoxy resin, resin transfer molding

## Abstract

In this work a holistic approach for the characterization and mathematical modeling of the reaction kinetics of a fast epoxy resin is shown. Major composite manufacturing processes like resin transfer molding involve isothermal curing at temperatures far below the ultimate glass transition temperature. Hence, premature vitrification occurs during curing and consequently has to be taken into account by the kinetic model. In order to show the benefit of using a complex kinetic model, the Kamal-Malkin kinetic model is compared to the Grindling kinetic model in terms of prediction quality for isothermal processing. From the selected models, only the Grindling kinetic is capable of taking into account vitrification. Non-isothermal, isothermal and combined differential scanning calorimetry (DSC) measurements are conducted and processed for subsequent use for model parametrization. In order to demonstrate which DSC measurements are vital for proper cure modeling, both models are fitted to varying sets of measurements. Special attention is given to the evaluation of isothermal DSC measurements which are subject to deviations arising from unrecorded cross-linking prior to the beginning of the measurement as well as from physical aging effects. It is found that isothermal measurements are vital for accurate modeling of isothermal cure and cannot be neglected. Accurate cure predictions are achieved using the Grindling kinetic model.

## 1. Introduction

Processing of highly reactive polymer materials is nowadays a key technology to achieve low cycle times and subsequently decrease manufacturing costs of both structural and non-structural parts. However, as processes and part geometries get more and more complex, the commonly adopted practice, involving many design iterations until a satisfactory result is obtained, becomes far too expensive. Therefore, it is preferable to experimentally characterize the reaction kinetics to enable model predictions and eventually optimize the process with the help of numerical simulation methods. The latter is even more important when fiber reinforced materials are used since the anisotropic behavior and the strong dependency of material properties on cross-linking makes the resulting part geometry and tolerances difficult if not impossible to predict.

Relevant effects which have to be considered are thermal expansion or contraction, chemical shrinkage and the evolution of material parameters as cross-linking advances. Furthermore, the actual material behavior is also dependent on cure temperature $T_{c}$ and glass transition temperature $T_{g}$. The ratio of these two temperatures determines whether the material is in glassy $\left(T_{c}\leq T_{g}\right)$ or rubbery $\left(T_{c}>T_{g}\right)$ state. As $T_{g}$ strongly depends on the degree of cure [1], it is very important to track this material property throughout the process. Otherwise it is not possible to determine the correct material state and consequently inappropriate material properties will be used by the simulation. This is especially important in processes with isothermal temperature programs, e.g., resin transfer molding (RTM), where the cure temperature is significantly lower than the ultimate glass transition temperature $T_{g,\infty }$. This results in premature vitrification before the maximum degree of cure $\alpha_{max}$ has been reached. In this case, the reaction has to take place in the glassy state where cross-linking either advances significantly slower than in rubbery state or, as observed in many cases, stops as the reaction rate completely vanishes [2,3,4]. Furthermore, important material properties undergo substantial changes as the resin vitrifies. Lange et al. [4] reported a change in thermal expansion and stiffness of an epoxy resin by as much as 51% and 260% respectively. In addition, O’Brien et al. [5] found relaxation to be very sensitive to the degree of cure, especially near gelation. This has huge impact on the development and relaxation of residual stresses during curing and cooling down which in turn affects the amount of warpage and the final shape of composite parts. Consequently, a corresponding process simulation method which aims at predicting this kind of manufacturing influences needs to take vitrification effects into account.

Various studies have been carried out using rather slow resin systems which have their primary field of application in small volume manufacturing as it is common e.g., in aerospace industry. These systems are much easier to analyze compared to highly-reactive resins which are commonly used by the automotive industry to satisfy requirements of large-batch production, particularly short cycle times. However, characterization of fast resins is more prone to errors especially under relevant isothermal conditions as these systems typically require rather high curing temperatures. This challenges common analyzing methods since the time lag at the beginning of a measurement needed for heating up to the desired temperature and thermal equalization may mask significant portions of the already ongoing cure reaction which is not recorded by the differential scanning calorimetry DSC analyzer [6]. As a result, some authors avoid isothermal measurements and concentrate solely on non-isothermal data. Spoelstra et al. [7] investigate the chemorheology of a filled epoxy system and declare isothermal DSC measurements to be inadequate in case of fast curing thermosets. Furthermore, they claim to be able to predict reaction kinetics for any temperature-history using their fitted model. Since the post-gelation region is not in their scope, this statement holds true but it does not necessarily in case of isothermal processes where premature vitrification cannot be neglected. Hsieh and Su [8] achieve accurate model predictions for isothermal curing using solely non-isothermal DSC data as long as vitrification does not occur. The same is reported by González-Romero and Casillas [3] where prediction accuracy impairs dramatically in case of vitrification. Moreover, they found isothermal measurements to be more usable for parameter estimation compared to non-isothermal although a higher number of measurements is required. Atarsia and Boukhili [9] convert non-isothermal cure data to isothermal and vice versa for the purpose of modeling the cure under non-uniform temperature profiles using an isoconversional representation. Again, the agreement between isothermal DSC and corresponding model predictions using only non-isothermal data is good as long as no vitrification occurs.

The above discussion shows that isothermal measurements are vital for proper modeling of isothermal processes which are affected by vitrification. Therefore, this study aims at demonstrating which experiments are needed for accurate modeling. Special attention is given to the evaluation of isothermal DSC measurements which suffer from unrecorded cross-linking at the beginning of the measurement. The latter was recently addressed by Javdanitehran et al. [10] who developed an iterative approach for characterization of isothermal curing kinetics. However, they focused on modeling cross-linking only until the reaction rate became negligibly small due to vitrification. Furthermore, although their predicted isothermal cure degree in general agrees well with experimental data, the slope towards the end of the cure curves is not reproduced accurately. As a consequence, incorrect cure degrees will be obtained for longer periods of time which is a major drawback when utilizing such predictions for cure cycle optimization. In the present study, this issue is solved by using the complete and uncut time ranges of the isothermal measurements in order to force the applied kinetic model to accurately reproduce diffusion-controlled reaction kinetics even for lengthy periods of time. However, achieving this goal is not only a question of having suitable measurements at hand. Choosing appropriate mathematical models is also an important requirement, though the present study is not intended to give a comprehensive benchmark on the multitude of available kinetic models. The comparison is therefore limited to two kinetic models: the Kamal-Malkin kinetic model [11], which is often applied in commercial process simulation software and the more advanced and complex Grindling kinetic model [12]. The latter is able to take into account premature vitrification which is crucial for accurate modeling of isothermal processes like RTM.

## 2. Experiments

### 2.1. Material

A commercial epoxy resin system typically used in resin injection processes was chosen for this study. It consists of two components: resin Biresin CR170 (Bisphenol-A-Epichlorohydrin) and hardener CH150-3 (3-Aminomethyl-3,5,5-trimethylcyclohexylamin and 2-Piperazin-1-ylethylamin), kindly provided by Sika Deutschland GmbH (Stuttgart, Germany). The recommended processing temperatures for this system range between 60 and 120 ${}^{\circ }$C. Batches of 10 g were mixed using a resin to hardener mixing ration of 100:24 (by weight). From these, the DSC specimens were obtained.

### 2.2. Characterization

Differential scanning calorimetry (DSC) is by far the most used method for cure kinetics characterization and thus is also used in the present study. DSC analyzers capture the exothermic heat flow which is released during the cross-linking process of a reactive polymer in respect of curing time under isothermal or non-isothermal conditions. Moreover, since the heat capacity of a polymer is sensitive to temperature and shows significant change during glass transition, the corresponding glass transition temperature $T_{g}$ can be measured. All DSC runs are carried out in nitrogen atmosphere and by using a DSC Q200 analyzer from TA Instruments (New Castle, DE, USA).

As has been implied above, non-isothermal DSC measurements are typically easier to perform because the reaction rate is virtually zero in the beginning if a sufficiently low start temperature is used. Therefore, the start temperature was set to −50 ${}^{\circ }$C for all non-isothermal DSC runs. This procedure of course is not applicable to isothermal measurements which therefore bear the risk of losing the first seconds or even minutes of cross-linking. Different strategies exist for minimization of this uncertainty. Slowly curing materials may be heated within the DSC furnace to the desired curing temperature. However, if the same is applied to fast curing resins, this can lead to a significant amount of cross-linking already before data acquisition starts since maximal controlled temperature ramps of typical DSC devices max out at 60 to 120 ${}^{\circ }$C/min. Alternatively, the specimen can be put into a preheated DSC furnace. In addition, the specimen may also be preheated prior to insertion into the DSC cell in order to reduce the effective temperature difference. Obviously, this leads to the same problem as before in case of fast curing resins. Therefore, in this study, the DSC cell was preheated to the respective isothermal temperature, but the specimens, which were obtained from a freshly mixed charge at room temperature, were not. In combination with small sample sizes and therefore fast temperature equalization between DSC cell and specimen, premature cross-linking is avoided as much as possible. In this study, specimens of about 7 and 10 mg were used. Even though, the assumption of zero degree of cure at the beginning of isothermal measurements at elevated temperatures is arguable.

#### 2.2.1. Reaction Kinetics

**Figure 1:**
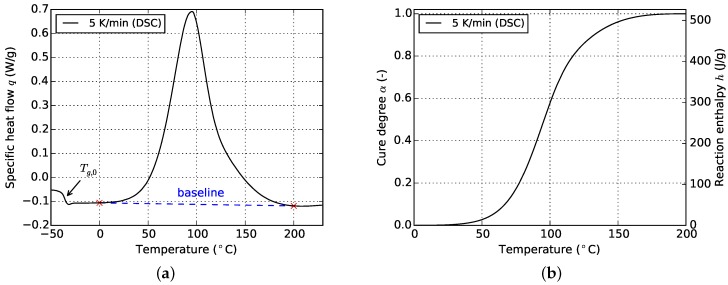
Evaluation of DSC data (exemplary for non-isothermal curing). (**a**) Specific heat flow; (**b**) Cure degree.

Various measurements were conducted in order to obtain a sufficient amount of data points for parametrization of the selected kinetic models. Both, isothermal and non-isothermal temperature programs are investigated in this study. The corresponding temperatures and heat rates are 60, 80, 100 and 120 ${}^{\circ }$C and 1, 2.5, 5, 10 and 15 ${}^{\circ }$C/min respectively. Results from the non-isothermal measurements are used for determination of the maximum total heat of reaction of the resin [13]. This value is a key quantity on which much of the later shown experimental analysis relies. It is derived by at first constructing a baseline for the reaction peak as is depicted in Figure 1.

The area enclosed by the specific heat flow curve q(t) and the baseline corresponds to the specific reaction enthalpy $h\left(t\right)$:

(1) $$h\left(t\right)=\int_{t_{0}}^{t}\;q\left(\hat{t}\right)\;d\hat{t}.$$

In this equation, $t_{0}$ denotes the time at which the reaction starts. Together with the specific total heat of reaction Δh, the temporal evolution of the cure degree $\alpha \left(t\right)$ is given by

(2) $$\alpha \left(t\right)=\frac{1}{\Delta h}\cdot h\left(t\right)=\frac{1}{\Delta h}\cdot \int_{t_{0}}^{t}\;q\left(\hat{t}\right)\;d\hat{t}.$$

**Figure 2:**
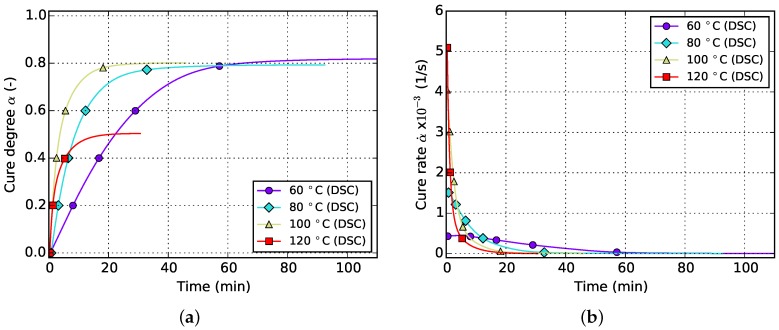
Data for isothermal curing at different temperatures derived from DSC measurements. (**a**) Cure degree; (**b**) cure rate.

**Figure 3:**
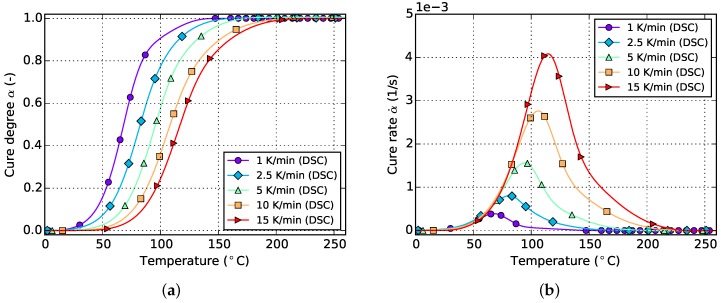
Data for non-isothermal curing at different temperature ramps derived from DSC measurements. (**a**) Cure degree; (**b**) cure rate.

The resulting curves for isothermal and non-isothermal curing are displayed in Figure 2 and Figure 3, respectively. The evolution of the cure degree *α* starts at 0 which represents the material in uncured state. During curing, it can reach a maximum value of 100% or 1, representing fully cured resin.

The aforementioned difficulty related to isothermal measurements is visible in Figure 2a. Usually, higher temperatures lead to higher cure degrees since $T_{g}$ can reach greater values before vitrification occurs and the reaction halts. However, the isothermal DSC measurements in Figure 2a do not show such a behavior. Instead a decrease in final cure degree is noticeable. This is in contradiction to the findings of Section 5.1 which prove higher achievable cure degrees for increasing isothermal temperatures as well as incomplete cure in case of lower curing temperatures even for long periods of time. This error is therefore attributed to the amount of cure which is lost during specimen preparation and measurement initialization. Additionally, as can be seen in Figure 2b, the cure rate strongly depends on temperature and, more importantly, reaches maximal values at the very beginning of the measurement. Therefore, even small periods of time without data acquisition in this region can introduce significant deviations in cure degree. This circumstance has to be addressed during model parametrization as otherwise poor prediction accuracy is to be expected.

The slope of isothermal cure curves vanishes for long periods of time as is visible in Figure 2a for all investigated temperatures. The reason for this is the influence of premature vitrification and the accompanied transition to diffusion-controlled reaction kinetics. Since this phenomenon depends mainly on the interaction of cure and glass transition temperature, this behavior is also observed for very low heating rates in non-isothermal DSC (cf. Figure 3a). In this case, the glass transition temperature increases faster as the cure temperature and finally outruns it, causing a slow down in reaction rate. As the cure temperature is further increased during non-isothermal DSC, the glass transition temperature also continues to rise and consequently prohibits the reaction from switching back to a chemical-controlled state. The effect is indicated by a flattening and almost constant slope of the curve in the affected cure range. For the studied epoxy resin, this happens during cure at the lowest heating rate of 1 ${}^{\circ }$C/min and for cure degrees above approximately 88%. However, the influence is much less pronounced. In particular, it does not show a complete stop in cross-linking as is shown by isothermal measurements.

The cure rate of non-isothermal experiments shows a dependency on temperature, as is visible in Figure 3b. However, in case of small heat ramps, most of the reaction takes place at rather low temperatures since the dwell time in this region is long enough to compensate for low temperatures. On the contrary, high heat ramps shift the majority of the reaction towards higher temperatures since these are reached in shorter periods of time. Corresponding curves of the cure degree confirm this behavior as can be seen in Figure 3a.

#### 2.2.2. Glass Transition Temperature

While characterization of reaction kinetics usually involves isothermal and non-isothermal DSC runs, measurement of $T_{g}$ at particular cure levels is conducted by combining both temperature programs. This procedure generally can be used instead of normal isothermal measurements as was previously shown by Fava [14]. The specimen firstly is hold isothermal using a specific time-temperature-combination. Afterwards, it is quickly cooled down far below the isothermal temperature (typically below 0 ${}^{\circ }$C) in order to fully stop cross-linking. Following this, it is heated up to a high temperature using a constant temperature ramp. Because of this temperature program, this type of measurement is referred to as cyclic DSC for simplicity in this study.

Determination of $T_{g}$ from DSC heating scans is generally not recommended since $T_{g}$ represents the temperature of transition from liquid/rubber to glass instead of the reverse [15]. However, DSC devices are usually calibrated on heating and, more importantly, the state of the material is inevitably altered by first heating the specimen to a temperature markedly above $T_{g}$ and then cooling down for the purpose of actual $T_{g}$ measurement. The reason for incorrect values gathered from heating experiments arises from commencing the glass transition from a possibly non-equilibrium state. This depends largely on the thermal history which was previously applied to the specimen. Therefore, reasonable values can be obtained by starting the heating scan immediately after cooling and by ensuring that curing prior to cooling down is carried out at a temperature $T_{c}>T_{g}$ such that no vitrification occurs [15,16].

Table 1 contains combinations of temperature and hold time used in this study to reach specific cure states. Afterwards, cooling of the specimen is carried out at the highest rate possible by the DSC analyzer (about 40 ${}^{\circ }$C/min). Thereby, further cross-linking during cooling down is minimized. Subsequent heating is realized by using a constant rate of 5 ${}^{\circ }$C/min for all cyclic measurements. The results are analyzed the same way as has been described above for non-isothermal DSC. However, since the cyclic measurement starts with an isothermal temperature program, it is subject to the same problems and errors like pure isothermal DSC runs. Therefore, the degree of cure is calculated reversely by evaluating the reaction peak of the non-isothermal part. Because of the preceding isothermal stage, the area of the non-isothermal reaction peak is generally smaller for cyclic DSC. This remaining heat of reaction is referred to as $\Delta h_{R}$. With this, the degree of cure reached at the end of the isothermal phase is calculated by

(3) $$\alpha =\frac{\Delta h-\Delta h_{R}}{\Delta h}=1-\frac{\Delta h_{R}}{\Delta h}.$$

## 3. Mathematical Modeling

Choosing an appropriate mathematical model for glass transition temperature as well as reaction kinetics is essential for getting meaningful results and predictions e.g., for process optimization. However, the field of application needs to be considered since the area of polymer processing includes a wide range of different temperature boundary conditions. This study concentrates on using the model for simulation of the resin transfer molding process which typically shows premature vitrification due to isothermal processing at rather low temperatures, as has been mentioned before.

In this section, model equations and corresponding fitting parameters are presented. Identification of the latter is described in Section 4.

### 3.1. Glass Transition Temperature

Vitrification of the resin starts once the glass transition temperature, which persistently rises during cross-linking, comes close to the current process temperature. Therefore, it is vital to have precise knowledge of the evolution of $T_{g}$ during the reaction. A widely used model, which is originally based on work of DiBenedetto [1,17], is given by

(4) $$\frac{T_{g}-T_{g,0}}{T_{g,\infty }-T_{g,0}}=\frac{\lambda \alpha }{1-\left(1-\lambda \right)\alpha }.$$

The glass transition temperature of the material in the uncured and fully cured state, $T_{g,0}$ and $T_{g,\infty }$ respectively, can be determined experimentally through DSC. Adopting the curve to experimental results is therefore done by adjusting the fitting parameter *λ* between 0 and 1.

### 3.2. Reaction Kinetics

Many different models for reaction kinetics exist. A comprehensive overview is given by Halley and Mackay [13]. From this variety, two models were selected which differ greatly in complexity.

#### 3.2.1. Kamal-Malkin Kinetic Model

As a simple autocatalytic model, the Kamal-Malkin kinetic model [11] is used. It is often applied and implemented in commercial process simulation software because of its simplicity and the relatively small number of model parameters which facilitates parametrization. The cure rate $\dot{\alpha }$ is defined by

(5) $$\dot{\alpha }=\frac{d\alpha }{dt}=\left(K_{1}+K_{2}\cdot \alpha^{m}\right)\cdot {\left(1-\alpha \right)}^{n}$$

where reaction rate constants $K_{1}$ and $K_{2}$ are given by Arrhenius-type equations

(6) $$K_{1}=A_{1}\cdot \exp\left(-\frac{E_{1}}{R\cdot T}\right)$$

(7) $$K_{2}=A_{2}\cdot \exp\left(-\frac{E_{2}}{R\cdot T}\right).$$

Besides the universal gas constant *R* and the temperature *T*, this kinetic model contains six fitting parameters: constants *m* and *n*, pre-exponential factors $A_{1}$ and $A_{2}$ as well as two activation energies denoted by $E_{1}$ and $E_{2}$. None of the model equations is able to account for vitrification effects. In combination with the term $\left(1-\alpha \right)$ on the right hand side of Equation (5), this model presumes the material to fully cure no matter what temperature is applied. Especially when using lower temperatures, this can lead to a significant loss in accuracy of the model prediction since the achievable degree of cure of resins is temperature dependent. However, since the Kamal-Malkin kinetic model is often used for process simulation, it is considered in this study in order to demonstrate the benefit of using a more complex model.

#### 3.2.2. Grindling Kinetic Model

In order to overcome the drawback of the Kamal-Malkin kinetic model, Grindling [12] extended the model towards taking into account diffusion controlled reaction kinetics. It is therefore a suitable choice for modeling isothermal molding processes and is included in this study in order to show the benefit from using a more sophisticated model.

Since the model was only published in German language, it is presented here in more detail. In a first step, Equation (5) is modified to

(8) $$\frac{d\alpha }{dt}=K_{1}\cdot {\left(1-\alpha \right)}^{n_{1}}+K_{\mathrm{eff}}\cdot \alpha^{m}\cdot {\left(1-\alpha \right)}^{n_{2}}.$$

The effective reaction rate constant $K_{\mathrm{eff}}$ enables the kinetic model to switch between a chemical-controlled and a diffusion-controlled state. It is defined by

(9) $$\frac{1}{K_{\mathrm{eff}}}=\frac{1}{K_{2,\mathrm{diff}}}+\frac{1}{K_{2}}.$$

In these two equations, $K_{1}$ and $K_{2}$ represent reaction rate constants which are only limited by chemistry. Therefore, both are modeled the same way as before (cf. Equations (6) and (7)). On the contrary, the newly introduced reaction rate constant $K_{2,\mathrm{diff}}$ represents a reaction rate that is limited by the diffusion velocity of the reactants and has therefore a strong dependency on the glass transition temperature $T_{g}$ and its distance to the current reaction temperature $\left(T-T_{g}\right)$:

$T>\left(T_{g}+\Delta T_{g}\right)$:
  (10) $$K_{2,\mathrm{diff}}=K_{2,\mathrm{diff}}\left(T=T_{g}+\Delta T_{g}\right)\cdot \exp\left(-\frac{E_{2,\mathrm{diff}}}{R}\cdot \left(\frac{1}{T}-\frac{1}{T_{g}+\Delta T_{g}}\right)\right)$$
$T_{g}\leq T\leq \left(T_{g}+\Delta T_{g}\right)$:
  (11) $$K_{2,\mathrm{diff}}=K_{2,\mathrm{diff}}^{T=T_{g}}\cdot \exp\left(\frac{c_{1}\cdot \left(T-T_{g}\right)}{c_{2}+T-T_{g}}\right)$$
$T<T_{g}$:
  (12) $$K_{2,\mathrm{diff}}=K_{2,\mathrm{diff}}^{T=T_{g}}\cdot \exp\left(-\frac{E_{1,\mathrm{diff}}}{R}\cdot \left(\frac{1}{T}-\frac{1}{T_{g}}\right)\right)$$

Equations (10) and (12) follow an Arrhenius-type approach with pre-exponential factors $K_{2,\mathrm{diff}}$ and $K_{2,\mathrm{diff}}^{T=T_{g}}$, as well as activation energies $E_{1,\mathrm{diff}}$ and $E_{2,\mathrm{diff}}$. Equation (11) is based on a modified Williams-Landel-Ferry (WLF) approach and introduces two parameters, $c_{1}$ and $c_{2}$, which enable the model to adjust the smoothness of the transition between the rubbery and the vitrified state. The activation energies $E_{1,\mathrm{diff}}$ and $E_{2,\mathrm{diff}}$ are calculated using both WLF-coefficients of Equation (11), $c_{1}$ and $c_{2}$:

(13) $$\frac{E_{1,\mathrm{diff}}}{R}=T_{g}^{2}\cdot \frac{c_{1}}{c_{2}}$$

(14) $$\frac{E_{2,\mathrm{diff}}}{R}=\frac{c_{1}\cdot c_{2}\cdot {\left(T_{g}+\Delta T_{g}\right)}^{2}}{{\left(c_{2}+\Delta T_{g}\right)}^{2}}$$

This kinetic model is considerably more complex than the previously shown Kamal-Malkin model. The eleven fitting parameters are: $A_{1},E_{1},n_{1},A_{2},E_{2},n_{2},m,c_{1},c_{2},K_{2,\mathrm{diff}}^{T=T_{g}}$ and $\Delta T_{g}$. In its original implementation [12], a fixed value of 100 ${}^{\circ }$C was used in place of the parameter $\Delta T_{g}$ without detailed explanation. However, $\Delta T_{g}$ has proven to be an important model parameter and therefore was added to the fitting algorithm in the present study.

## 4. Parameter Identification

Identifying appropriate model parameters is crucial for precise kinetic modeling. However, due to the phenomenological character of the used models, it is difficult to estimate even the order of magnitude of the fitting parameters beforehand. As a consequence, the resulting search space to cover is very large. Therefore, the evolutionary algorithm covariance matrix adaptation evolution strategy (CMA-ES) introduced by Hansen and Ostermeier [18] is utilized in this study.

The curve fitting algorithm attempts to reproduce the measured values by using the chosen model. As is shown in the next section, the prediction accuracy of the fitted model depends on the type of experimental DSC data handed over to the algorithm as target values. The DSC results are categorized with respect to the type of measurement: non-isothermal, isothermal and cyclic. In order to evaluate which type of measurement is beneficial for obtaining good fitting results, parameter estimation is performed by using either solely one of these measurement categories or a combination of them.

The aforementioned problem of non-zero cure degree at the beginning of DSC data acquisition in case of isothermal measurements is counteracted by calculating the initial amount of cross-linking iteratively by using the current model parameter set. It is assumed that the missing part of the reaction can be reconstructed during parameter estimation using the kinetic model. Considering that in this case the initial cure value of the isothermal curves from both, experiment and model, can vary widely during fitting, it is necessary to include another measurement type which contributes fixed values of cure degree, like cyclic or non-isothermal DSC. In other words, it is not advisable to fit a model by solely using isothermal data since all curves will level out at full cure (α=1) after fitting due to the variability in initial cure. Otherwise, fitting to solely isothermal data under negligence of α>0 at the beginning of the measurement is neither reasonable since then the model needs to handle the non-physical situation of lower final cure in spite of higher isothermal curing temperature as is visible in Figure 2.

## 5. Results and Discussion

### 5.1. Glass Transition Temperature

The glass transition temperature $T_{g}$ of the resin at intermediate cure states is evaluated from cyclic DSC measurements as is outlined in Section 2.2.2. For calculation of the corresponding cure degrees, the total heat of reaction Δh is determined from non-isothermal measurements. A mean value of Δh = 499.8 ± 16.6 J/g is found for the studied epoxy resin. Moreover, a mean glass transition temperature of the fully uncured resin of $T_{g,0}$ = −30.64 ${}^{\circ }$C is determined from non-isothermal DSC runs as it is indicated in Figure 1b. For the fully cured resin, a glass transition of $T_{g,\infty }$ = 135.74 ${}^{\circ }$C is measured by immediately reheating a specimen which was previously cured to *α* = 100%. During this second heating, $T_{g,\infty }$ becomes visible.

**Figure 4:**
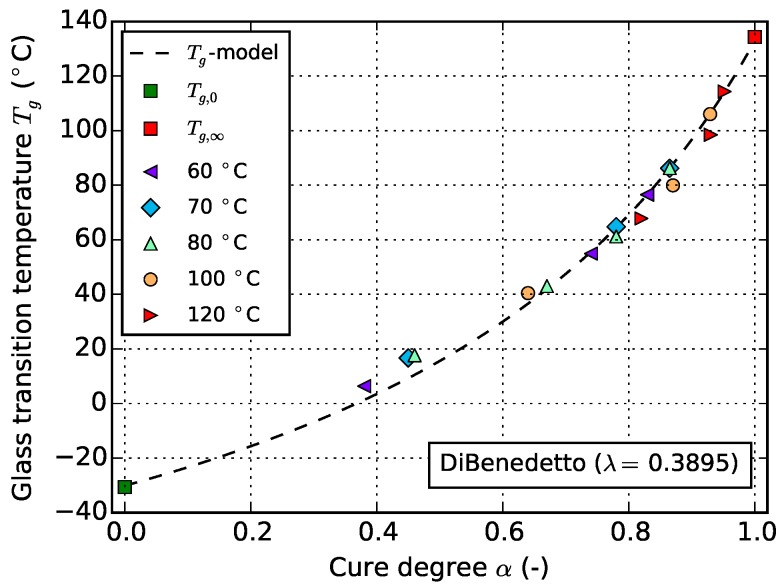
Dependency of $T_{g}$ on cure degree *α*: experimental values determined from cyclic DSC according to Table 1, compared to the prediction of the fitted DiBenedetto model.

Figure 4 shows the dependency of $T_{g}$ on cure degree as well as the fitted DiBenedetto $T_{g}$-model which is in good agreement with experiments. The corresponding value for the model parameter of Equation (4) is *λ* = 0.3895. The experimental values are determined by creating the midpoint between the glassy and the liquid/rubbery lines and identifying the corresponding temperature. Because of the different hold times and curing temperatures, the achieved cure degrees differ.

From an experimental point of view, using only a single low isothermal temperature together with a large number of different hold times for characterization of $T_{g}\left(\alpha \right)$ would be favorable since high temperatures, which introduce errors in the first seconds, are avoided. However, because of premature vitrification at low temperatures, this is not possible. Moreover, the material exhibits physical aging effects when it is hold for long time at a specific temperature after reaching the maximal degree of cure possible at that temperature [19]. These effects show up during the second stage of cyclic DSC runs and can make correct determination of $T_{g}$ and $\Delta h_{R}$ difficult if not impossible. Consequently, it is recommended to use $T_{g}$ values determined from measurements which do not exhibit vitrification (valid values) [1]. This is the case for all but the longest times at each temperature (corresponding times are highlighted in Table 1).

**Figure 5:**
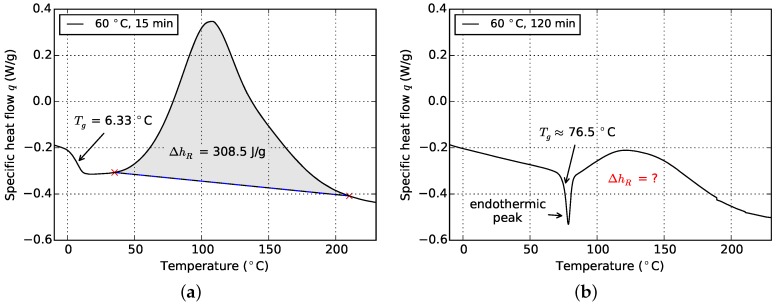
Temperature scanning data after isothermal curing at 60 ${}^{\circ }$C for different periods of time: influence of physical aging on data evaluation. (**a**) Cured at 60 ${}^{\circ }$C for 15 min; (**b**) cured at 60 ${}^{\circ }$C for 120 min.

Figure 5 shows the influence of physical aging on the evaluation of data from cyclic DSC measurements. The specimen which was cured at 60 ${}^{\circ }$C for 15 min (Figure 5a) allows for good distinction between the glass transition and the onset of the reaction peak. On the contrary, in case of the specimen which was cured at the same temperature but was hold for 120 min (Figure 5b), these events are superimposed by an endothermic peak which araises from physical aging. Construction of a baseline and subsequent integration in order to determine *α* is no longer possible. However, since it is an advantage for later cure model fitting to have values of *α* and the corresponding $T_{g}$ also in vitrified material state available, an alternative strategy is pursued. In a first step, a $T_{g}$-model is fitted using only valid values of $T_{g}$ and *α*. In this case, this is done using the model of DiBenedetto (cf. Section 3.1). From this relationship, the cure degrees which cannot be determined from cyclic DSC due to physical aging effects are derived reversely by using associated $T_{g}$ values. The latter is possible since from the mentioned three overlapping effects, glass transition is the best-preserved one. Thus determination of $T_{g}$ is possible (cf. Figure 5a), although the values might be affected by physical aging. However, the error of *α* values gathered that way is compensated to some extent by the non-linear behavior of $T_{g}\left(\alpha \right)$ towards high cure degrees (cf. Figure 4). Furthermore, because $T_{g}$ gains in sensitivity with respect to cross linking in this region, it is even more suited for monitoring cross-linking than *α* [20].

### 5.2. Reaction Kinetics

One major goal of this study is to find out which measurements need to be conducted in order to achieve good prediction accuracy. In particular, the benefit of incorporating data from cyclic DSC measurements, which are necessary anyhow for characterization of the relationship between the glass transition temperature $T_{g}$ and the cure degree *α*, is demonstrated by firstly discussing results fitted without this data. Afterwards, the same is done under consideration of cyclic data in order to compare both cases.

For reasons of clarity and comprehensibility, the layout of the presentation of fitting results, consisting of three graphs placed side by side, is kept constant throughout the discussion. On the left graph, the predicted isothermal curing is plotted against time. The plot in the middle shows the model-prediction for non-isothermal temperature profiles and on the right, the consistence with cyclic DSC measurements is evaluated. The latter plot contains additional black lines which show the discrepancy between the prediction and the experimental data point.

While each figure contains plots for all three measurement types, the types that were actually used for curve fitting vary (cf. Section 4). This way, it is possible to visualize how good a kinetic model is able to reproduce each of the measurement types although not all of them may have been actually considered during fitting. However, one exception is formed by isothermal DSC data. Since the initial degree of cure cannot be predicted when isothermal measurements are not used for fitting of the model, it is not feasible to plot all of the corresponding experimental curves in such a way that it is possible to evaluate the prediction quality. Hence, only the experimental data of the lowest temperature (60 ${}^{\circ }$C) is plotted because in this case, the amount of cure which is initially lost is insignificant.

#### 5.2.1. Prediction Accuracy without Using Cyclic DSC Measurements

**Figure 6:**
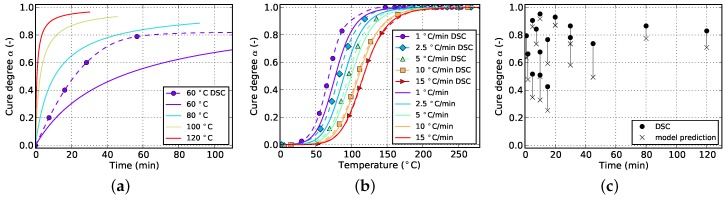
Cure prediction of Kamal-Malkin model fitted solely to non-isothermal data. (**a**) Isothermal; (**b**) non-isothermal; (**c**) cyclic.

**Figure 7:**
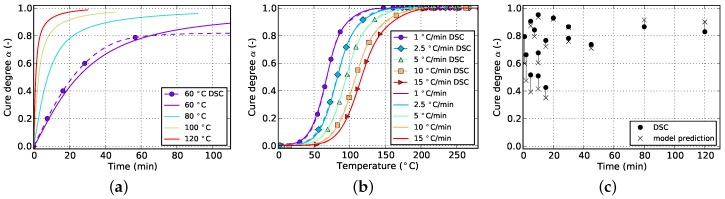
Cure prediction of Grindling model fitted solely to non-isothermal data. (**a**) Isothermal; (**b**) non-isothermal; (**c**) cyclic.

Figure 6 and Figure 7 show the goodness of fit for both kinetic models in case only non-isothermal DSC data is used. As can be seen from the non-isothermal plots, the Grindling model is able to reproduce the experimental data very closely whereas the Kamal-Malkin model shows a significant deviation. From the plot on the right (Figure 7c) it can be seen that the Grindling model shows good accuracy also for the isothermal conditions during cyclic DSC. However, the slope at the end of the curves on the isothermal plot (Figure 7a) is overestimated and does not exhibit slowing down of cross-linking due to vitrification (cf. Figure 2).

**Figure 8:**
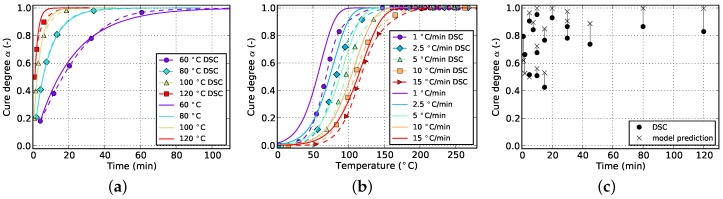
Cure prediction of Kamal-Malkin model fitted solely to isothermal data. (**a**) Isothermal; (**b**) non-isothermal; (**c**) cyclic.

**Figure 9:**
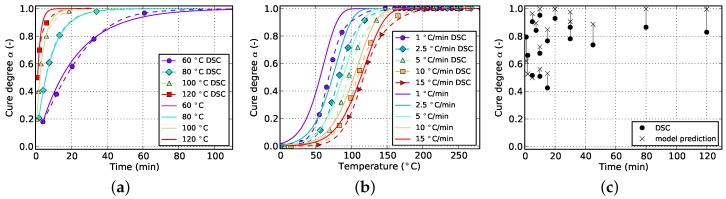
Cure prediction of Grindling model fitted solely to isothermal data. (**a**) Isothermal; (**b**) non-isothermal; (**c**) cyclic.

In Section 4 it was concluded that fitting to only isothermal data is not advisable. Figure 8 and Figure 9 show the result of the fitting algorithm for this situation for Kamal-Malkin and Grindling kinetic model, respectively. Obviously, prediction accuracy of both models is poor which is especially evident from the comparison with cyclic DSC data. The algorithm overpredicts the initial cure degree such that all isothermal curves eventually end up at full cure $\left(\alpha =1\right)$. As a consequence, the reaction never has to take place under diffusion-controlled conditions. This leads to very similar cure predictions since the Grindling model is an extension of the Kamal-Malkin model and behaves similar in the absence of vitrification (cf. Section 3.2). Therefore, as has been mentioned before, using only isothermal data for kinetic model fitting is not sufficient.

**Figure 10:**
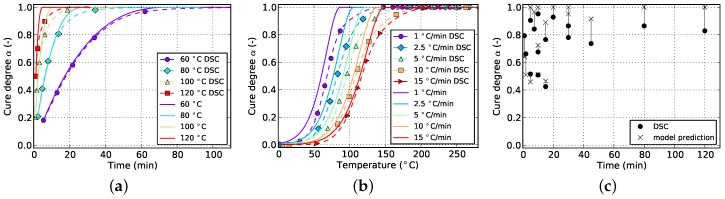
Cure prediction of Kamal-Malkin model fitted to isothermal and non-isothermal data. (**a**) Isothermal; (**b**) non-isothermal; (**c**) cyclic.

**Figure 11:**
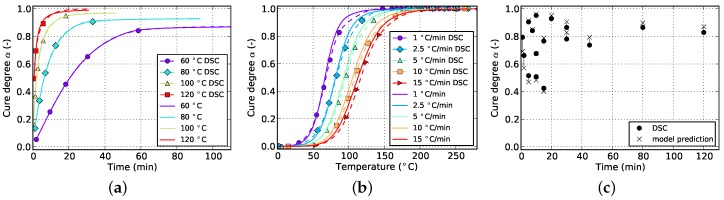
Cure prediction of Grindling model fitted to isothermal and non-isothermal data. (**a**) Isothermal; (**b**) non-isothermal; (**c**) cyclic.

By using non-isothermal as well as isothermal data as the fitting target, it is possible to iteratively calculate the initial cure degree for the experimental isothermal curves. When comparing the corresponding plots in Figure 10 and Figure 11, the advantage of diffusion-controlled kinetics in case of the Grindling kinetic model becomes obvious. The Kamal-Malkin model is not able to reproduce both isothermal and non-isothermal data in a sufficient way. From Figure 10a it is visible that the fitting algorithm chooses the initial cure degrees such that all curves reach a cure level of 100%. This was to be expected since the model is not capable of modeling vitrification effects. This model actually delivered much better prediction accuracy for non-isothermal and isothermal processing when only non-isothermal data was used for fitting (cf. Figure 6). The Grindling model shows very good prediction accuracy for both temperature programs due to its ability to adjust the cure rate not only to cure temperature but also to the current glass transition temperature.

#### 5.2.2. Prediction Accuracy Using Cyclic DSC Measurements

Up to this point, kinetic model fitting was carried out without using data from cyclic DSC measurements. However, when using diffusion-controlled kinetic models, these measurements need to be performed anyway in order to characterize the dependency of $T_{g}$ on cross-linking. It may therefore be beneficial to also use them for fitting of kinetic models.

**Figure 12:**
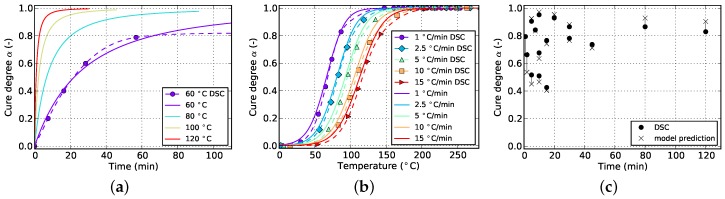
Cure prediction of Kamal-Malkin model fitted to non-isothermal and cyclic data. (**a**) Isothermal; (**b**) non-isothermal; (**c**) cyclic.

**Figure 13:**
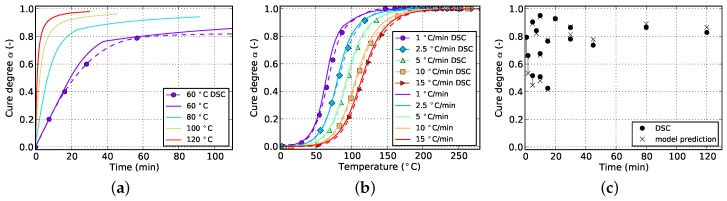
Cure prediction of Grindling model fitted to non-isothermal and cyclic data. (**a**) Isothermal; (**b**) non-isothermal; (**c**) cyclic.

By comparing Figure 6 and Figure 12, it becomes evident that by adding cyclic data to the fitting target, the prediction accuracy of the Kamal-Malkin kinetic model for isothermal processing is improved considerably. This is especially evident when comparing the match with cyclic DSC measurements (cf. Figure 6c and Figure 12c). However, the slope at the end of isothermal curves still remains too steep. The reason for that is the type of data which is gathered from cyclic DSC runs. In contrast to pure isothermal measurements, cyclic DSC measurements as they are evaluated in the present paper, only provide data at distinct points. More specifically, there is no information about how a specific cure degree was reached or for how long a possible vitrification might have been present. As can be seen in Figure 7 and Figure 13, the Grindling model shows slightly better accuracy after adding cyclic data to the fitting target. Moreover, a sudden transition from chemically-controlled to diffusion-controlled reaction is indicated by a kink in the curve for isothermal cure at 60 ${}^{\circ }$C in Figure 13a.

In general, it is observed that isothermal cure prediction gains in accuracy by adding cyclic data to the fitting target, which apart from that only consists of non-isothermal data. At the same time, prediction quality for non-isothermal conditions decreases when applying the Grindling model (cf. Figure 7b and Figure 13b). This was to be expected since the model now has to not only reproduce non-isothermal curing, but also, at least to some extent, isothermal processing in the form of cyclic DSC. The opposite is observed in case of the Kamal-Malkin model, which shows a slight improvement in non-isothermal cure predictions, particularly for low heat rates (cf. Figure 6b and Figure 12b).

**Figure 14:**
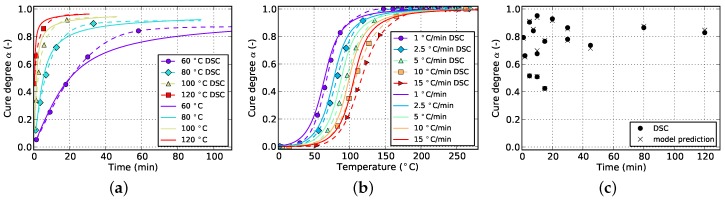
Cure prediction of Kamal-Malkin model fitted solely to isothermal and cyclic data. (**a**) Isothermal; (**b**) non-isothermal; (**c**) cyclic.

**Figure 15:**
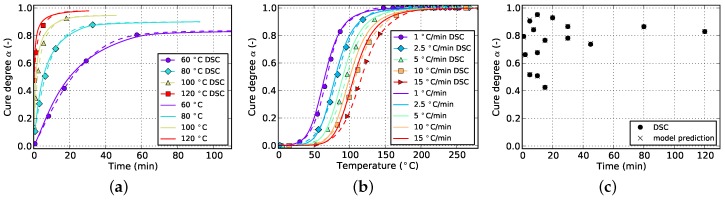
Cure prediction of Grindling model fitted to isothermal and cyclic data. (**a**) Isothermal; (**b**) non-isothermal; (**c**) cyclic.

When fitting to solely isothermal data, the match between experimental and numerical curves improves significantly by additionally incorporating cyclic data into the fitting target. This is visible in Figure 8 and Figure 14 for the Kamal-Malkin kinetic model and in Figure 9 and Figure 15 for the Grindling kinetic model. However, the gain is more pronounced in case of the latter model.

The Kamal-Malkin model compensates for premature vitrification under isothermal conditions by significantly slowing down the reaction after reaching medium high cure levels (cf. Figure 14a). The match with cyclic DSC data is therefore good but even though the reaction is slowed down, it will reach full cure for longer periods of time. Moreover, vitrification occurs for distinct isothermal temperatures at different cure levels which the Kamal-Malkin kinetic model is not able to account for. This results in a too early slow down of the reaction in case of the lowest as well as a too late slow down for the highest isothermal temperature. Hence, the glass transition temperature which is directly related to the cure degree is underestimated, or in the latter case, overrated. This can have a significant impact when using such predictions for process simulation since many material properties depend on the glass transition temperature and whether the material is in glassy or rubbery state.

Although none of the non-isothermal experimental data is used for fitting both models give good predictions for non-isothermal curing. The accuracy is best for the lowest heating rate which is due to the fact that the isothermal measurements provide many data points for widespread values of cure degree within a temperature range of 60 to 120 ${}^{\circ }$C. Most of the reaction of specimens which are cured at a heating rate of 1 ${}^{\circ }$C/min, happens within this temperature range (cf. Figure 14c and Figure 15c) and therefore the model is able to accurately predict the curing process. The agreement between model prediction and experiment declines as the heating rate is increased because the reaction is shifted towards higher temperatures which require the kinetic models to extrapolate the temperature dependency of the cure rate into this region. This introduces deviations which are most evident in case of the highest heating rate of 15 ${}^{\circ }$C/min (cf. Figure 14b and Figure 15b).

**Figure 16:**
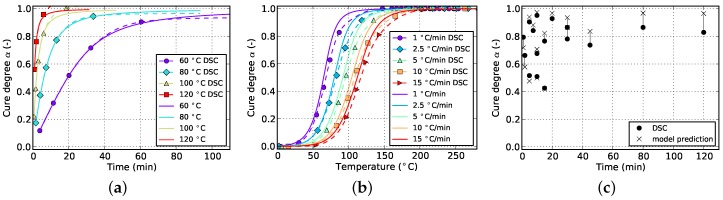
Cure prediction of Kamal-Malkin model fitted to isothermal, non-isothermal and cyclic data. (**a**) Isothermal; (**b**) non-isothermal; (**c**) cyclic.

Figure 10 and Figure 16 show the difference between fitting to non-isothermal as well as isothermal and doing so with additionally using cyclic data in case of the Kamal-Malkin kinetic model. An improvement is visible for each measurement type. However, isothermal cure predictions still end up at too high values of cure degree, which again arises from the fact, that the model is not able to render vitrification effects. The slow down in cure rate, which was observed when fitting against isothermal and cyclic data (cf. Figure 14a), is less pronounced in this case. This is due to a change in weighting of each of the measurement types. If fitting is carried out under consideration of only isothermal and cyclic data, vitrification is present in all of the experimental data. However, this is not the case for non-isothermal data and by adding this type of measurement to the fitting process, data with vitrification loses in weight.

**Figure 17:**
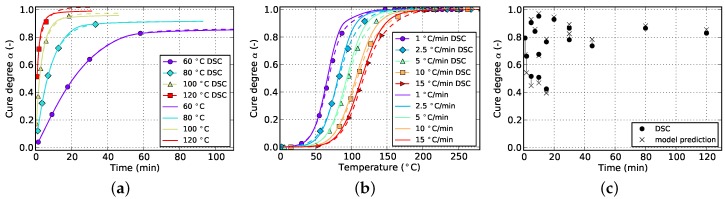
Cure prediction of Grindling model fitted to isothermal, non-isothermal and cyclic data. (**a**) Isothermal; (**b**) non-isothermal; (**c**) cyclic.

On the contrary, by comparing the results of the Grindling model in Figure 11 and Figure 17, a very good match between experiment and model is observed regardless of whether cyclic data is used for fitting or not. The slight deviations may also arise from the fitting algorithm itself. However, by comparing Figure 15b and Figure 17b, it is observed that by adding non-isothermal data to the fitting target, the prediction accuracy for this type of processing is improved whereas at the same time, the match with cyclic data loses in quality. This is again because of the fact that the model now has to satisfy a more comprehensive situation.

In order to simplify the comparison of the different fitting results, the goodness of fit is quantified by standard errors, as summarized in Table 2. In this table, non-isothermal and isothermal DSC measurements are abbreviated as dyn and iso, respectively. Lower values of standard error denote a better accordance between measurement and model prediction. However, it is important to mention that the prediction quality for isothermal conditions is overrated when the initially lost amount of cross-linking is overestimated by the fitting algorithm. This is observed for both models when fitted against solely isothermal data, and in case of the Kamal-Malkin model when fitted to isothermal and non-isothermal data. On the contrary, the prediction accuracy for isothermal curing is underrated when isothermal measurements are not used for model fitting, since the initial cure degrees cannot be estimated under this circumstances. In this case, the standard error is calculated with the assumption of zero initial cure (cf. Figure 2a). Corresponding values of standard error are marked accordingly in Table 2. Due to the mentioned restrictions, the validity and comparability of the shown standard errors is limited. Interpretation of the data in Table 2 should therefore always be accompanied by visual comparison of corresponding cure predictions.

**Figure 18:**
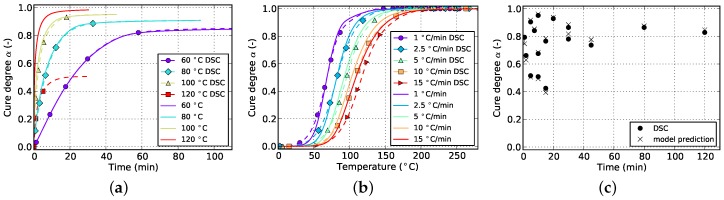
Cure prediction of Grindling model fitted to cyclic data and a reduced set of isothermal and non-isothermal experiments: 60, 80 and 100 ${}^{\circ }$C (isothermal) and 1, 5 and 15 ${}^{\circ }$C/min (non-isothermal). (**a**) Isothermal; (**b**) non-isothermal; (**c**) cyclic.

Since it is favorable to reduce the needed amount of measurements, Figure 18 shows the fitting quality of the Grindling kinetic model in case cyclic data and only a reduced set of isothermal and non-isothermal measurements are used for model parametrization. The isothermal data is limited to the temperatures 60, 80 and 100 ${}^{\circ }$C. In case of non-isothermal DSC, only results for heating rates of 1, 5 and 15 ${}^{\circ }$C/min are used.

By comparing Figure 17a and Figure 18a only minor differences can be identified. This suggests that isothermal measurements at high temperatures close to the ultimate glass transition temperature $T_{g,\infty }$ can be omitted. This may be due to the fact that at this elevated temperatures, the influence of vitrification is much smaller compared to lower temperatures. Since DSC data of isothermal curing at 120 ${}^{\circ }$C was not used for fitting, a corresponding initial degree of cure was not predicted. Therefore, the experimental curve in Figure 18a shows far too low cure degrees.

Prediction quality for non-isothermal processing (cf. Figure 17b and Figure 18b) loses in accuracy. However, this is acceptable since for the majority of composite manufacturing processes, i.e., RTM, good and accurate results for isothermal temperature programs is much more important. Furthermore, the match with cyclic data is improved (cf. Figure 17c and Figure 18c) which again is advantageous for modeling of isothermal processes.

It should be clear from the above that the often applied approach of only using non-isothermal DSC measurements for kinetic model parametrization is not suitable for prediction of isothermal curing at temperatures below the ultimate glass transition temperature. Instead, the results of the present study indicate that the non-isothermal measurements are actually not as important as the isothermal ones. This is due to the fact that isothermal measurements contain information of both, cross-linking with and without influence of vitrification. Non-isothermal DSC shows this effect only for very low heating rates, for which the glass transition temperature is able to catch up with the reaction temperature. This results in a temporarily decrease in reaction rate. However, since the temperature is continuously increased, non-isothermal DSC data does not contain information about a complete stop in reaction as is observed under isothermal conditions. Using isothermal measurements for model parametrization, however, requires the fitting strategy to account for the initially lost amount of cure which cannot be avoided when conducting DSC measurements of fast resins at elevated isothermal temperatures. By iteratively approximating the initial cure degrees during fitting, as presented in this study, this major drawback of isothermal DSC runs was successfully eliminated.

## 6. Conclusions

In this study, the prediction accuracy of a simple (Kamal-Malkin) and a complex (Grindling) kinetic model was investigated and compared to each other for non-isothermal, isothermal and cyclic temperature programs. The Grindling model was chosen because of its ability to account for premature vitrification, which typically occurs during isothermal processes like RTM. In this context, the cure dependency of the glass transition temperature was characterized and a non-linear behavior of the investigated epoxy resin towards high cure degrees was observed. Furthermore, since some cyclic DSC measurements showed enthalpy relaxation due to physical aging, a strategy was presented which enables derivation of reliable cure degree values from this data with the aid of a parametrized $T_{g}$-model. This additional values proved to be beneficial for subsequent fitting of the kinetic models.

Comparison of the goodness of fit of both kinetic models was carried out for parameter sets which were fitted using various combinations of the three measurement types. It was shown that for obtaining accurate and reliable cure predictions for non-isothermal or isothermal processing, it is generally necessary to include corresponding measurements in the fitting process. It is especially not sufficient to use only non-isothermal data for fitting of a model which is intended to predict isothermal curing at temperatures below the ultimate glass transition temperature. Instead, it is vital to include measurements into the fitting process which contain information concerning relevant effects like vitrification. However, using solely isothermal data also led to poor results regardless of the applied kinetic model due to fundamental problems closely related to specimen preparation and DSC data acquisition in general. A strategy was presented in order to use this type of measurement for model fitting despite of the mentioned problems by iteratively approximating the initial amount of cure which was not covered by the DSC analyzer. However, as has been shown, this approach is only of advantage if additional data from non-isothermal or cyclic DSC runs is available during fitting. Consequently, very good accuracy for isothermal processing was achieved when fitting the complex Grindling model against isothermal and cyclic data. The simpler Kamal-Malkin model also benefits from this approach. However, prediction quality for isothermal curing is inferior to the results of the Grindling model. Non-isothermal curing is predicted by both models with good results although none of the corresponding measurement data was used for fitting. Compared to fitting by using solely non-isothermal data, the mean error for both models is reduced by about 60%. An equal reduction in prediction error is achieved when using isothermal and non-isothermal data, although in this case good results were only achieved by the Grindling kinetic model.

Predictions of the Kamal-Malkin kinetic model are generally inferior to predictions of the Grindling model. When using the Grindling model, the standard error can be reduced by a maximum of 65% compared to the Kamal-Malkin model.

In relation to the question which measurements are required for accurate cure modeling in isothermal processes, it is important to make clear that this can only be accomplished by a suitable kinetic model. As has been shown in this study, the simpler Kamal-Malkin kinetic model is not able to account for vitrification effects which are important in isothermal processing. As a result, it generally fails in accurately predicting the evolution of the cure degree under isothermal conditions. The more complex Grindling model achieved significantly better results but necessitates the characterization of the evolution of the glass transition temperature during cross-linking. However, since cure modeling is often done in the context of process simulation, this relationship needs to be determined anyway, e.g., for the purpose of modeling the evolution of mechanical parameters during cross-linking. Furthermore, results of this study indicate that having isothermal and cyclic data available obviates the need for conducting non-isothermal DSC runs. However, the total heat of reaction Δh, which is needed for evaluation of the DSC data, as well as the ultimate glass transition temperature $T_{g,\infty }$ were determined in this study from non-isothermal data. It is therefore not possible to completely disregard non-isothermal DSC runs but a decrease in number is feasible. Moreover, results of this study indicate that isothermal measurements at temperatures near the ultimate glass transition temperature can be omitted. This way, very good prediction accuracy is achieved by at the same time reducing the experimental effort.

## Figures and Tables

**Table 1:** Parameters of cyclic DSC runs.

Curing temperature $T_{c}$ (°C)	Hold times $t_{h}$ (min)
60	15, 45, 120 *
70	10, 30, 80 *
80	5, 10, 15, 30 *
100	2, 7.5, 20 *
120	1, 5, 10 *

* Vitrification was observed during isothermal curing.

**Table 2:** Prediction accuracy (standard error) of the kinetic models parametrized using different fitting targets (lower values denote better accuracy).

Kinetic model	Prediction case	Fitting target					
		dyn *	iso	dyn-iso	dyn-cyclic	iso-cyclic	dyn-iso-cyclic
Kamal-Malkin	**isothermal**	0.1634 ${}^{‡}$	0.0230 ${}^{†}$	0.0393 ${}^{†}$	0.1614 ${}^{‡}$	0.0642	0.0177
	**non-isothermal**	0.0337	0.0734	0.0693	0.0311	0.0655	0.0430
	**cyclic**	0.1521	0.1061	0.1193	0.0580	0.0119	0.0684
	mean error	0.1164 ${}^{‡}$	0.0675 ${}^{†}$	0.0760 ${}^{†}$	0.0835 ${}^{‡}$	0.0472	0.0430
Grindling	**isothermal**	0.1360 ${}^{‡}$	0.0230 ${}^{†}$	0.0087	0.1496 ${}^{‡}$	0.0519	0.0089
	**non-isothermal**	0.0040	0.0734	0.0258	0.0194	0.0520	0.0258
	**cyclic**	0.0874	0.1061	0.0457	0.0541	0.0038	0.0524
	mean error	0.0758 ${}^{‡}$	0.0675 ${}^{†}$	0.0267	0.0744 ${}^{‡}$	0.0359	0.0290

* dyn = non-isothermal DSC (dynamic temperature scanning); ${}^{†}$ initial cure degree is overestimated (no premature vitrification during isothermal curing); ${}^{‡}$ initial cure degrees assumed to be zero because isothermal DSC data is not used for fitting (cf. Figure 2a).

## Abbreviations

The following abbreviations are used in this manuscript:

DSC	Differential Scanning Calorimetry
RTM	Resin Transfer Molding
CMA-ES	Covariance Matrix Adaptation Evolution Strategy
